# Engineering the next-generation of CAR T-cells with CRISPR-Cas9 gene editing

**DOI:** 10.1186/s12943-022-01559-z

**Published:** 2022-03-18

**Authors:** Alexander Dimitri, Friederike Herbst, Joseph A. Fraietta

**Affiliations:** 1 Department of Microbiology, Perelman School of Medicine, University of Pennsylvania, South Pavilion Expansion (SPE), Room 9-104, 3400 Civic Center Blvd, Bldg. 421, Philadelphia, PA 19104-5156 USA; 2 Center for Cellular Immunotherapies, Perelman School of Medicine, University of Pennsylvania, Philadelphia, PA USA; 3 Abramson Cancer Center, Perelman School of Medicine, University of Pennsylvania, Philadelphia, PA USA; 4 Department of Translational Medical Oncology, National Center for Tumor Diseases, Dresden and German Cancer Research Center, Heidelberg, Germany; 5 Department of Pathology and Laboratory Medicine, Perelman School of Medicine, University of Pennsylvania, Philadelphia, PA USA

**Keywords:** CRISPR, CAR T-cell, Gene editing, Immunotherapy, Cancer

## Abstract

Chimeric Antigen Receptor (CAR) T-cells represent a breakthrough in personalized cancer therapy. In this strategy, synthetic receptors comprised of antigen recognition, signaling, and costimulatory domains are used to reprogram T-cells to target tumor cells for destruction. Despite the success of this approach in refractory B-cell malignancies, optimal potency of CAR T-cell therapy for many other cancers, particularly solid tumors, has not been achieved. Factors such as T-cell exhaustion, lack of CAR T-cell persistence, cytokine-related toxicities, and bottlenecks in the manufacturing of autologous products have hampered the safety, effectiveness, and availability of this approach. With the ease and accessibility of CRISPR-Cas9-based gene editing, it is possible to address many of these limitations. Accordingly, current research efforts focus on precision engineering of CAR T-cells with conventional CRISPR-Cas9 systems or novel editors that can install desired genetic changes with or without introduction of a double-stranded break (DSB) into the genome. These tools and strategies can be directly applied to targeting negative regulators of T-cell function, directing therapeutic transgenes to specific genomic loci, and generating reproducibly safe and potent allogeneic universal CAR T-cell products for on-demand cancer immunotherapy. This review evaluates several of the ongoing and future directions of combining next-generation CRISPR-Cas9 gene editing with synthetic biology to optimize CAR T-cell therapy for future clinical trials toward the establishment of a new cancer treatment paradigm.

## Background

The utility of CRISPR-Cas9 technology has led to a surge in applying genome editing approaches to combat a variety of genetic disorders and cancers. In these cases, inherited genetic diseases with known gene mutations can be corrected [1, 2]. This technology can also be applied to induce specific mutations in the setting of in vitro and in vivo experimental models of disease to study various interventional strategies. In the context of T-cell-based immunotherapies, applications of CRISPR-Cas9 technology are being explored to improve T-cell effector function and persistence, reduce treatment-related toxicity, and increase patient product availability [3, 4].

## Main text

### Overview of chimeric antigen receptor T-cell therapy and resistance mechanisms

**Fig. 1:**
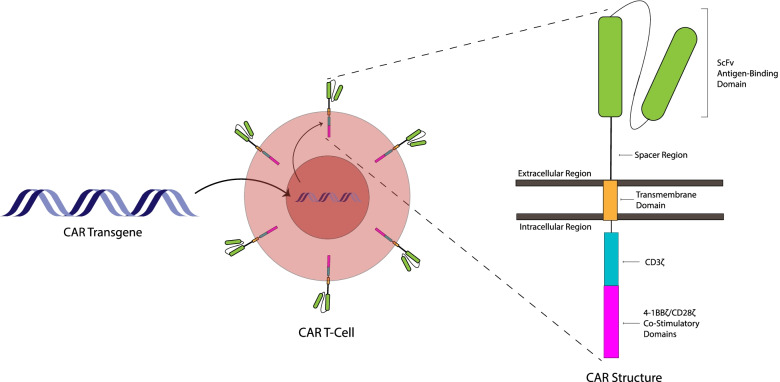
Schematic representation of a chimeric antigen receptor (CAR) T-cell. CAR T-cells are typically produced by transducing T-lymphocytes with a transgene encoding a synthetic antigen receptor. This transgene is integrated into the T-cell genome, transcribed and translated into a CAR protein. The core functional components of a CAR are binding and signaling domains separated into extracellular and intracellular compartments, respectively. The extracellular binding portion of the receptor is typically comprised of a single-chain variable fragment derived from the variable regions of an antibody that recognizes specific tumor antigens, together with a spacer that provides flexibility to the binding domain. The transmembrane domain connects the binding domain with intracellular signaling moieties. The TCR-derived CD3ζ chain drives T-cell activation and is fused in tandem with co-stimulatory endodomains that allow for robust and sustained function

CAR T-cell therapy has led to sustained remissions in populations of otherwise refractory patients and, more specifically, has demonstrated complete response rates of > 80–97% in certain B-cell malignancies such as acute lymphoblastic leukemia (ALL) [5, 6]. Because of this clinical efficacy, preclinical development of CAR T-cell therapy for several other cancer types is an area of active investigation, and the future of cellular immunotherapy will undoubtedly be extensive and dynamic. In the setting of conventional CAR T-cell therapy, a patient’s own T-cells are genetically engineered to express a synthetic receptor that recognizes a specific target antigen on tumor cells. The specificity and activation of the CAR T-cells typically result from combining extracellular antigen-binding domains from antibodies (e.g., single-chain variable fragments, scFvs; heavy-chain variable domain (VHH)-based binders) with the intracellular signaling machinery of the T-cell receptor (TCR) CD3ζ chain. Additional co-stimulation is provided in tandem with the signaling domains of molecules such as CD28 or 4-1BB to further potentiate T-cell activation and persistence (Fig. 1) [7–9]. The first United States Food and Drug Administration (FDA)-approved applications of CAR T-cell therapy occurred in 2017 with the release of the commercial CD19-directed CAR T-cell therapies Kymriah (tisagenlecleucel) and Yescarta (axicabtagene ciloleucel) that are used to treat B-cell ALL and diffuse large B-cell lymphoma (DLBCL) [7, 10, 11]. While CD19 is a transmembrane glycoprotein continuously and stably expressed on all stages of B-cell lineage differentiation and is thus present on healthy as well as malignant B-cells, aplasia resulting from CD19 CAR T-cell administration is clinically tolerated and made feasible through immunoglobulin replacement therapy [12]. Since then, other CD19 CAR T-cell products, as well as the first non-CD19 directed CAR targeted to B-cell maturation antigen (BCMA) for the treatment of multiple myeloma, have been approved [10, 13–15]. Additionally, numerous clinical trials are currently underway to assess the safety and efficacy of CAR products for hematopoietic malignancies and non-hematopoietic cancers. These next-generation products incorporate single antigen binders, tandem CARs and co-expressed receptors against multiple tumor targets, logic-gated receptors, and engineered delivery of payloads to enhance effector T-cell function [10, 16].

Despite these developments, the causes of unsuccessful CAR T-cell therapy are multifactorial and may not be addressed with synthetic biological improvements alone. Major limitations to successful CAR T-cell therapy occur both during the manufacturing process in vitro and during CAR T-cell proliferation in vivo. In many B-cell malignancies, complete responses are associated with robust CAR T-cell proliferation, with a clear advantage of long-term CAR T-cell persistence [6, 17–21]. Thus, limited CAR T-cell expansion and persistence following therapy is a common mechanism of treatment failure. Lack of therapeutic levels of in vivo CAR T-cell proliferation and persistence can be attributed in part to the pre-existing exhausted state of baseline or manufactured T-cells (e.g., in chronic lymphocytic leukemia [CLL]), acquisition of CAR T-cell hypofunction following infusion [20, 22–24], a reduction in stem cell memory/central memory differentiation [20, 25–28] and premature senescence [23]. There are also many patients who cannot benefit from this therapy because of issues with collecting their autologous T-cells, especially from subjects receiving intensive chemotherapy and with low numbers of memory T-cells that possess optimal proliferative capacity [20, 26].

Additional hurdles are specific to solid tumors where CAR T-cells must traffic to tumor sites and surmount stromal barriers to infiltrate the tumor bed and elicit tumor-specific cytotoxicity. Even if trafficking and infiltration are successful, T-cells can become dysfunctional due to a toxic tumor microenvironment (TME) characterized by metabolic perturbations, the presence of inhibitory soluble factors and cytokines, as well as elevated frequencies of suppressive immune cells or tumor cells that secrete these mediators and overexpress ligands for negative immune checkpoint receptors. Malignant cells also undergo antigen loss or downregulation to escape and evade CAR T-cell recognition at the tumor site. Potential CAR T-cell therapy patients may also experience advanced disease progression during the prolonged process of gaining enough quality T-cells for manufacturing. The above issues cannot be addressed with a single approach. However, many of these challenges may be overcome by using site-specific genetic editing in combination with next-generation cellular engineering approaches. With the genesis of easily multiplexable precision genome editing using clustered regularly interspaced short palindromic repeats (CRISPR)-CRISPR-associated protein 9 (Cas9) (CRISPR-Cas9) technology [29], there is an opportunity to circumvent many of these barriers to CAR T-cell therapy of cancer and fast-track integration of this treatment approach into routine medical management of a variety of malignancies.

### CRISPR-Cas9 gene editing modalities

**Fig. 2:**
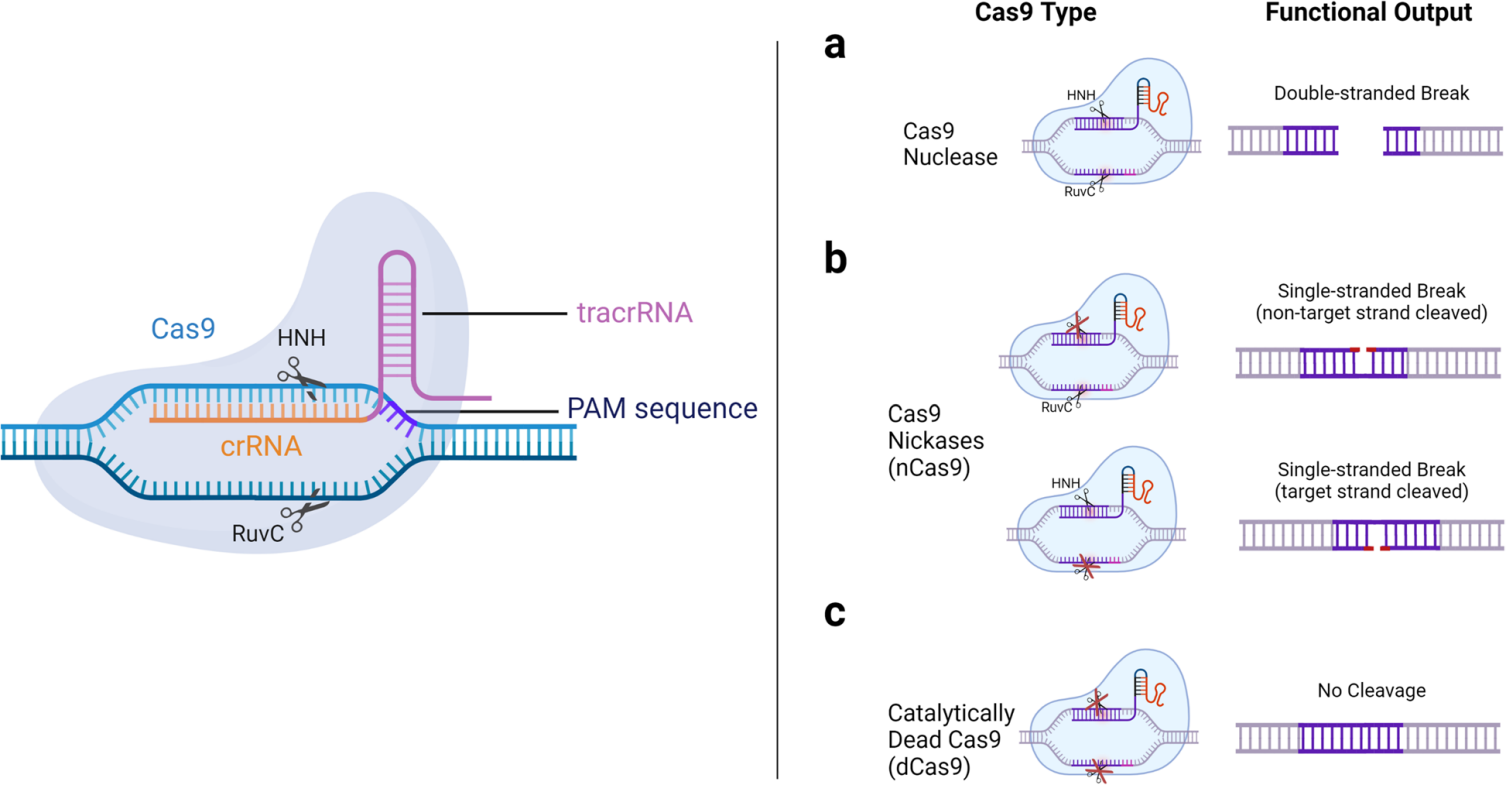
CRISPR/Cas9 components and Cas9 enzyme class variants. (Left) Endonuclease Cas9 can cleave a specific site within DNA as determined by the complementarity of the synthetic (crispr) crRNA to the target DNA strand, together with a (trans-activating) tracrRNA hairpin to provide structural stability. The crRNA and tracrRNA make up a single guide RNA (sgRNA) complex. Additional specificity is provided by the protospacer adjacent motif (PAM) that directs Cas9 to cut 3 base pairs upstream. **a** Cas9 cuts DNA via its HNH and RuvC domains that each cut a single DNA strand, resulting in a double-stranded break. **b** Mutations in one domain or both domains restrict(s) Cas9 to perform single-strand nicking (nCas9) or (**c**) to possess no catalytic activity (dCas9). Created with BioRender.com

The emergence of CRISPR-Cas9 technology with its simplicity, flexibility, and effectiveness has considerably improved the process and time frame of gene editing. Briefly, the Cas9 DNA endonuclease enzyme can be directed to virtually any site in the genome to create a double-stranded DNA break. The cleavage region for Cas9 is selected based on a 20-base pair single guide RNA sequence (sgRNA) that directs Cas9 to the target DNA cut site, which possesses sequence complementarity to the sgRNA. This site is additionally specified by a protospacer adjacent motif (PAM) sequence within the target DNA downstream of the cleavage site, consisting of any variation of 5’-NGG. Together with the core sgRNA, there are additional structures and RNA features like loops and hairpins needed for active and stable complex formation with Cas9 as detailed in Fig. 2 [30]. This site-specific DNA endonuclease system was first discovered in bacteria as a form of an ‘adaptive immune response’ to combat infections [31, 32]. Since then, investigators have exploited the core components of CRISPR-Cas9 to create site-specific gene edits in animal and human cell systems for a wide variety of applications (reviewed in [33]).

#### CRIPSR-Cas9 can be used for gene disruption and to regulate gene expression

**Fig. 3:**
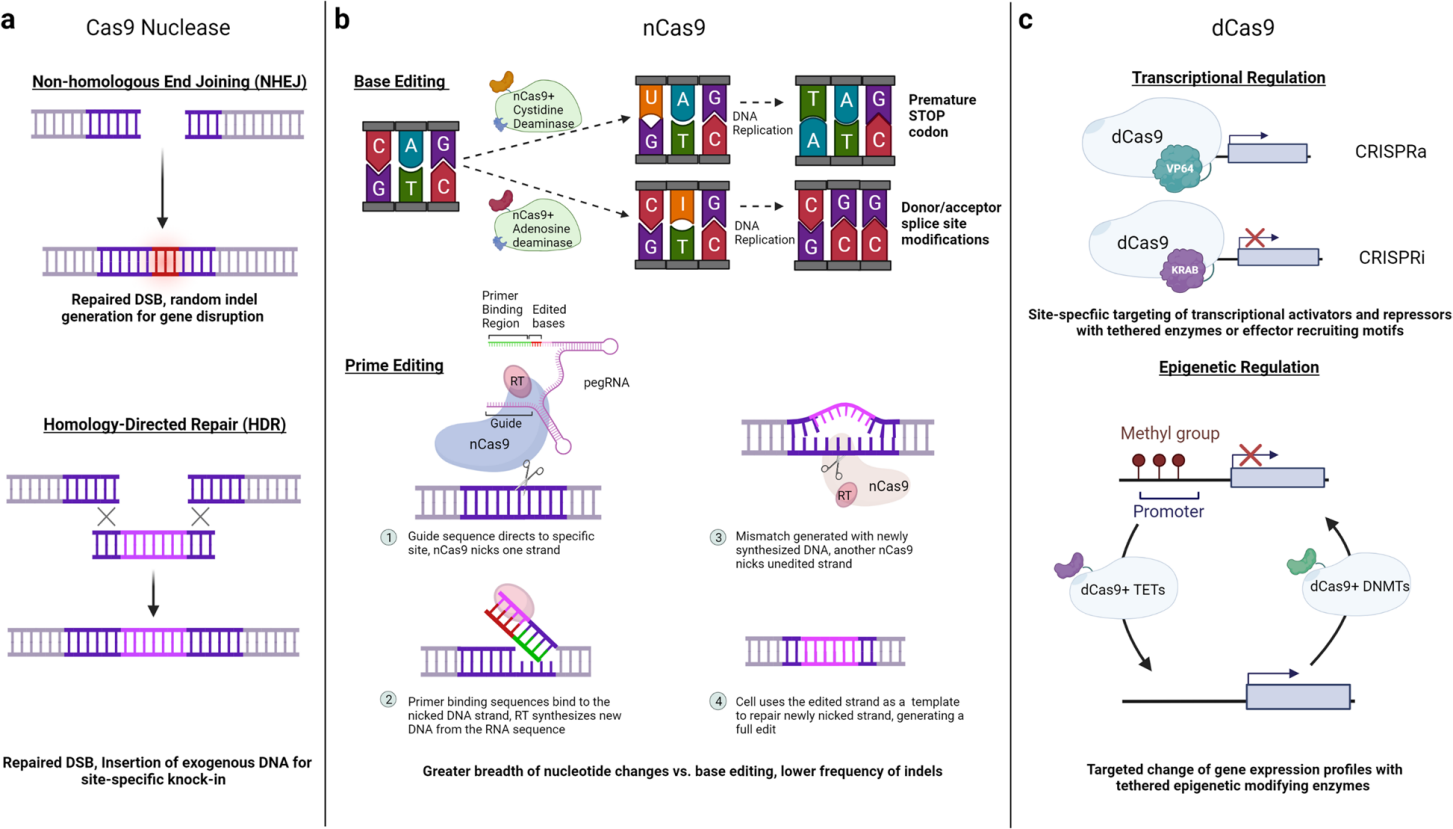
Applications of Cas9 variants. **a** Double-stranded DNA breaks (DSBs) generated by the Cas9 nuclease will be repaired by non-homologous end joining (NHEJ) or homology-directed repair (HDR) within the cell. During NHEJ, the ligase frequently adds random insertions and deletions (indels) to repair the break site. This process is considered as error-prone and used to cause generalized gene disruptions, typically with a loss-of-function outcome. HDR uses a repair template that has homology with sites upstream and downstream of the cut site, allowing for recombination of the template for an error-free repair. For gene editing purposes, an exogenous donor DNA repair template can be designed to insert large foreign DNA constructs into the site, allowing for site-specific knock-in that can result in reduction or gain of activity at the desired locus, or replacement with a new gene cassette. **b** nCas9 can be tethered to cytidine and adenosine deaminases to allow for single-base pair editing. Cytidine deaminase catalyzes the conversion of CT while adenosine deaminase will catalyze the conversion of AG. Base editing can be used to cause gene disruption without generating DSBs by altering coding regions to introduce premature STOP codons or to interfere with splicing donor/acceptor sites. nCas9 can also be fused to reverse transcriptase (RT), together with a specialized guide RNA (pegRNA) forming a complex that can be used to introduce larger sequence additions into a DNA region without creating DSBs. **c** Enzymatically dead Cas9 (dCas9) can be tethered to transcriptional activators (e.g., VP64) to potentiate transcription at a specific promotor region targeted by the gRNA, while transcriptional repressors (e.g., KRAB) can attenuate transcriptional activity. dCas9 can be tethered to epigenetic modifying enzymes to catalyze demethylation (e.g., TETs) or active methylation (DNMTs) for temporal regulation of epigenetic motifs and transcriptional activity at gRNA-specified loci promoters. Created with BioRender.com

The conventional Cas9 protein consists of six domains, REC I, REC II, Bridge Helix, PAM-Interacting, HNH and RuvC [34, 35]. Rec I is the largest domain and mediates guide RNA binding. The role of the REC II domain has not yet been well-characterized. The arginine-rich Bridge Helix is critical for inducing cleavage activity upon DNA binding [35]. Initiation of binding to target DNA is mediated by the PAM-Interacting domain that confers PAM specificity [9, 34–36]. The Cas9 nuclease component contains the HNH and RuvC nuclease domains that cleave both the target and non-target strands of DNA, generating a double-stranded break (DSB) (Fig. 2a). Introduction of a DSB will induce either non-homologous end joining (NHEJ) or homology-directed repair (HDR) to correct the break (Fig. 3a). NHEJ occurs during all cell cycle phases and is considered error-prone since it can introduce random base pair insertions and deletions (indels), resulting in a pool of edited cells that can be clonally selected for gene knockout. Alternatively, co-delivery of a transgene template, where the template is flanked by homology arms upstream and downstream of the cut site, enhances high-fidelity homologous recombination repair leading to an insertion of the desired transgene into the target locus, which is restricted to G2/S phases of the cell cycle. The transgene insertion can consist of anything from an expression marker (e.g., fluorescent molecule), a selectable reporter, an element that will up- or down-regulate target gene expression, or an entirely new gene cassette [37].

#### Different Cas9 delivery methods and enzyme variants can limit off-target editing

DSB induction permits gene disruption and/or transgene insertion into target loci. However, there are risks associated with such breakage when it occurs at off-target sites, potentially introducing unintended genome alterations. Thus, precise control of when and where DSBs occur is beneficial for reducing the frequencies of unintended indels and translocations [38] and preventing selection of p53 inactivated cells [39, 40]. It has been demonstrated that direct delivery of a pre-formed Cas9/gRNA ribonucleoprotein (RNP) complex into cells allows for Cas9 to be active immediately. The RNP is also quickly degraded once internalized, and therefore decreasing the amount of time Cas9 is present for potential off-target cleavage, but enough time to still maintain an optimal threshold of on-target editing efficiency [41–43].

Several Cas9 enzyme variants have also been developed to overcome the disadvantages of DNA DSB cleavage. For example, Nickase Cas9 (nCas9) variants contain mutations in either the RuvC or HNH domain which render Cas9 capable of cleaving only the targeted or non-targeted DNA strand, respectively [44] (Fig. 2b). The use of two pairs of nCas9/gRNA complexes flanking a targeted site allows for greater control of where a DSB can occur, as both complexes need to be at the same cut site for inducing the DSB, allowing for reduced off-target DSB generation [45]. Mutations in both catalytic domains create a catalytically dead Cas9 (dCas9) variant (Fig. 2c). dCas9 can easily be tethered to other functional enzymes to elicit a variety of site-specific modifications, independently of Cas9 catalytic activity [46].

#### Base editors can be used to introduce single base pair transitions

In the process of base editing (Fig. 3b), introduction of DNA point mutations can create or repair single nucleotide variants, modify donor and acceptor splicing sites, alter codon composition to change an amino acid code or introduce a premature STOP codon [47–49]. Accordingly, base editors can introduce four transition mutations that substitute a pyrimidine for another pyrimidine or purine for another purine (CT, TC, GA, AG) [48]. The enzymes used to catalyze these reactions are cytidine and adenosine deaminases. Cytidine deaminase induces the deamination of cytosine bases to uracil. During DNA replication, DNA polymerase ‘reads’ uracil as thymine, as these bases have the same base-pairing properties, resulting in a functional cystine-to-thymine correction in the nascent DNA strand. Adenosine deaminase acts in a similar manor, catalyzing the deamination of adenosine to inosine (I), which is ‘read’ by DNA polymerase as a guanine [50]. Base editing acts within a small window of specificity when directed to a precise site via a guide RNA (i.e., 13–18 base pairs upstream of the PAM site) [48]. To avoid off-target cuts [51–55] while achieving maximal editing efficiency, several Cas9 and deaminase variants have been developed that exhibit different PAM specificities [56–58] or editing windows [59–61]. Initial base editors relied on dCas9, but more recent investigations demonstrated that nCas9 is preferred for higher editing efficiency, as it can nick the non-edited strand to encourage the C-U or AI modified strand to be used as the template during DNA repair, resulting in a fully preserved edit [50]. Cytidine deaminase base editors are commonly used to generate premature STOP codons during transcription [48]. Finally, adenosine deaminase base editors can be used to alter splice donor/acceptor sites.

#### Prime editors can be applied to introduce transversions and deletions or insertions

A major limitation of base editing is that it cannot be applied to introduce transversion mutations (i.e., pyrimidine to purine and vice versa) as well as small deletions or insertions. To address this constraint, an alternative technology known as ‘prime editing’ was developed to facilitate all possible base-to-base conversions and targeted insertions or deletions without inducing DSBs [62]. In this strategy, nCas9 is tethered to an engineered reverse transcriptase (RT) enzyme and a prime editing guide RNA (pegRNA) directs this nCas9/RT complex to the desired genome region. In addition to the target recognition site, the pegRNA contains a binding region that associates with the nicked DNA strand serving as a primer, followed by an RNA sequence encoding the new template. RT synthesizes DNA from the pegRNA sequence onto the nicked DNA strand for further ligation. This creates a DNA mismatch, which can be corrected using a second nCas9/guideRNA complex (PE3 editing strategy). After nicking the unedited strand, the cellular DNA repair machinery corrects this lesion using the initial pegRNA-edited strand as a template, which leads to the full establishment of the desired gene edit at the target locus (Fig. 3b). Prime editors are capable of not only inducing base pair resolution substitutions, but also insertions of up to 44 base pairs and deletions as high as 80 base pairs [63]. Thus, like base editors, prime editors can be used to modify post-mitotic cells and demonstrate a reduced risk for genotoxicity that often accompanies the generation of multiple DSBs. Current strategies modulating the enzyme activity [64, 65] or the pegRNA structure have been undertaken to further improve the efficiency of prime editing [54, 66].

#### Catalytically dead Cas9 fused to transcriptional modulators can be used to regulate locus-specific gene expression

dCas9 can be used to promote or attenuate transcriptional activity based on recruitment of transcriptional activators or repressors to specific sites in the processes of CRISPR activation (CRISPRa) and CRISPR interference (CRISPRi), respectively [67–70] (Fig. 3c). CRISPRi incorporates a tethered transcriptional repressor such as a Krüppel associated box (KRAB) domain to induce repression of transcription at the specified transcriptional start site [46, 71]. Alternatively, CRISPRa involves tethering of transcriptional activators such as VP64 to promote endogenous gene expression [46, 72]. In addition, tethering demethylating ten-eleven translocation (TET) methylcytosine dioxygenases or DNA methyltransferases (DNMTs) can permit locus and region-specific epigenetic modifications [73, 74] (Fig. 3c). This strategy has previously been used to target hypermethylated promoter regions of tumor-suppressor genes [75].

### Prospects for improving CAR T-cell Therapy with CRISPR-Cas9 gene editing

#### CRISPR-Cas9-mediated gene editing can be exploited to ameliorate CAR T-cell dysfunction

**Fig. 4:**
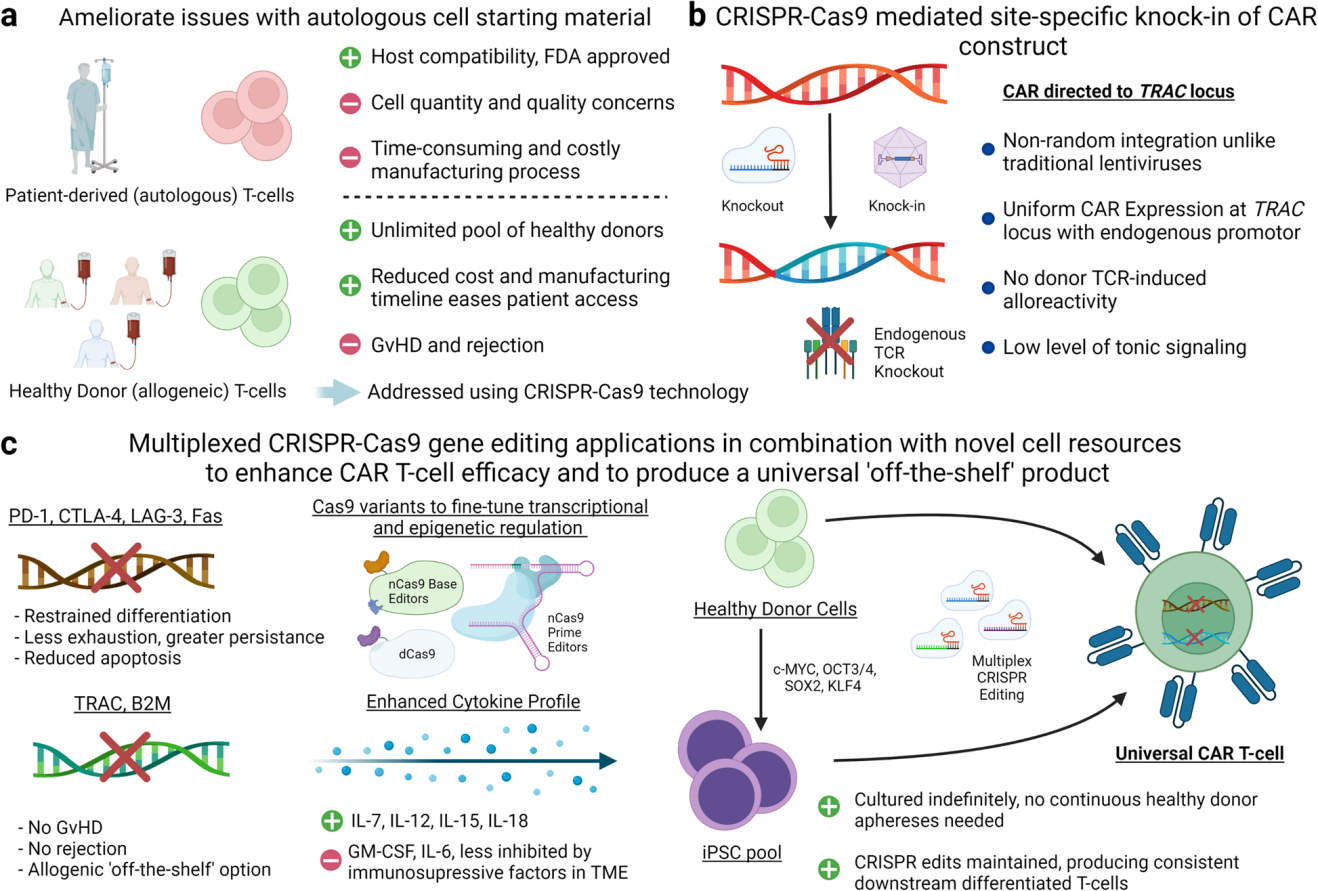
Summary of CRISPR-Cas9 editing strategies to generate optimally potent and widely available CAR T-cell products. **a** CRISPR-Cas9 editing can be used to develop allogeneic CAR T-cell therapies, which will ameliorate many of the current issues associated with autologous CAR T-cell products. **b** Removal of the endogenous TCR by targeting *TRAC* via CAR transgene knock-in addresses histocompatibility barriers associated with third party cell products derived from unrelated donors. **c** Multiplex CRISPR-Cas9 editing can be used to enhance the antitumor efficacy and improve the safety of autologous or allogeneic CAR T-cell products. CRISPR-Cas9-mediated precision editing of clonal master iPSC lines has the potential to generate a renewable cell source that can be repeatedly used to mass produce homogeneous, optimally potent, ‘best-in-class’ universal CAR T-cell products in a cost-effective manner. Created with BioRender.com

Numerous factors often collectively prevent durable remissions following CAR T-cell therapy, including autologous CAR T-cell manufacturing issues, limited CAR T-cell expansion and/or persistence as well as various T-cell-intrinsic and -extrinsic resistance mechanisms (Fig. 4a). Recent studies indicate that chronic exposure to high levels of antigen leads to a state of T-cell exhaustion. Tumors, by providing a persistent source of antigen while avoiding clearance, promote T-cell exhaustion [76]. Checkpoint blockade has been a successful approach to sustain T-cell function, and inhibitors that target negative T-cell regulators such as CTLA-4, PD-1, LAG-3, and TIM-3 are being tested in clinical trials to prevent or ameliorate exhaustion [76]. The mechanisms that lead to exhaustion in the setting of CAR T-cell therapy are complex and remain poorly understood. Our studies in patients with relapsed/refractory CLL treated with CD19 CAR T-cells identified the presence or absence of T-cell exhaustion signatures at the time of apheresis, done to harvest T-cells for cell manufacturing, as a major predictor of clinical outcome [20].

Several approaches have been taken to improve the survival and function of CAR T-cells, including optimization of costimulatory endodomains [77], prevention of tonic signaling [78], inhibition of NR4A transcription factors [79], TOX/TOX2 blockade [80] and overexpression of the canonical AP-1 factor, c-Jun [81]. Recently, global analysis of histone modifications in T-cells has elucidated marked differences in the chromatin structure of distinct subsets of exhausted T-cells and their transcriptional regulators (reviewed in [82]). Weber and colleagues [83] recently demonstrated that transient cessation of antigen receptor signaling through forced downregulation of the CAR protein or inhibition of proximal CAR signaling kinases restores functionality in exhausted T-cells through global and site-specific chromatin remodeling. Because intermittent CAR T-cell ‘rest’ mitigates exhaustion and enhances antitumor potency in association with epigenetic reprogramming, engineering loss-of-function or modulation of genetic and epigenetic targets that potentiate T-cell exhaustion using CRISPR-Cas9 technology has the potential to prevent and perhaps even reverse CAR T-cell dysfunction. Targeting inhibitory receptors, transcription factors and/or other mediators of CAR T-cell dysfunction through gene editing may induce reinvigoration of infused cell products. Removing negative regulators of T-cell persistence and effector function (e.g., PD-1, CTLA-4 and LAG-3) using CRISPR-Cas9 may indeed represent a first obvious tractable point of intervention [84–87]. In addition to inhibitory receptors, disruption of Fas receptor/Fas ligand interactions have been shown to reduce activation-induced cell death and potentiate increased in vivo CAR T-cell antitumor function [88]. Finally, CRISPR/Cas9-mediated targeting of diacylglycerol kinase (DGK) metabolism in CAR T-cells renders them resistant to immunosuppressive mediators in the TME, such as TGFβ [89]

#### CRISPR/Cas9-mediated cytokine modulation potentiates CAR T-cell function and reduces toxicity

Genetic strategies to modulate cytokine signaling during CAR T-cell activation and expansion have the potential to bolster antitumor activity, enhance T-cell persistence and/or reduced toxicity. CRISPR-Cas9 systems that incorporate transgene knock-in approaches have been exploited for this purpose. More specifically, CRISPR/Cas9-based gene editing combined with viral or non-viral DNA delivery permits simultaneous bi-allelic or sequential gene targeting to engineer T-cells with expression cassettes in a site-specific manner [90, 91] (Fig. 4b). Using this technology, cytokine-encoding DNA cassettes can be knocked into targeted genomic loci placing these genes under the control of specific promoters for temporal control of expression. For example, IL-15 has been knocked into the IL-13 gene locus, thus placing IL-15 expression under control of the endogenous IL-13 promoter, which is highly active upon T-cell activation. This creates an inducible T-cell specific IL-15 activation switch [92]. Additionally, removal of genes encoding cytokines that drive neurotoxicity and cytokine release syndrome (CRS) such as GM-CSF [93] and IL-6 [94] with CRISPR-Cas9 editing has the potential to produce an optimally potent and durably persistent cell product, while reducing adverse events associated with aberrant cytokine production. Accordingly, GM-CSF knockout CAR T-cells maintain normal functions and increased antitumor activity in vivo, and potentiate improved overall survival, compared to conventional CD19 CAR T-cells [93]. Genetic knockdown or ablation of the IL-6 gene also has the potential to ameliorate CRS-like toxicity in leukemia-bearing mice [95] (Fig. 4c).

#### CRISPR-Cas9 technology can be applied to knocking-in CAR cassettes

Many current CAR T-cell manufacturing protocols involve ex vivo autologous T-cell expansions followed by transduction with a viral vector containing the chimeric receptor sequence [96]. While lentiviral transduction and integration is stable and considered generally safe for clinical trials and FDA-approved treatments [97], there are possible risks for malignant transformation of engineered CAR T-cells via insertional mutagenesis of tumor suppressor genes or oncogenes [98]. Additionally, since lentiviruses integrate semi-randomly in the genome, the CAR transgene can insert in sites with high or low relative transcriptional activity, leading to variable cell-surface CAR expression, generating a sub-optimal therapeutic product [99]. Placing the CAR transgene under control of a strong exogenous promoter may also lead to high constitutive receptor expression. Elevated surface expression and interaction with other CAR receptors can generate ligand-independent tonic signaling in the absence of exogenous signal. This can induce both systemic production of cytokines as well as a cell profile that drives rapid transition to poor effector function and T-cell exhaustion [100, 101]. To address these caveats associated with lentiviral transduction, CRISPR-Cas9 can be used to deliver a CAR-encoding DNA cassette to a specific genomic location, allowing for targeted knock-in of the CAR into desired sites. For example, an anti-CD19 CAR can be directed to the T-cell receptor α constant (*TRAC*) locus, resulting in uniform CAR expression, reduced tonic signaling, decreased exhaustion and increased antitumor efficacy [90] (Fig. 4b) Targeting *TRAC* also gives the added benefit of producing a potential universal product.

### Application of CRISPR-Cas9 in Universal CAR T-cell product generation

Clinical development of novel CAR T-cell therapies is often hampered by the low yield and poor functionality of mature, autologous peripheral blood T-cells from many elderly and heavily-pretreated patients. A proposed solution is the procurement of healthy donor leukocytes to produce ‘universal’ T-cells with optimized in vivo persistence and antitumor potency. However, generating off-the-shelf CAR T-cell products is challenging because multiple genome edits within finite numbers of differentiated T-cells are needed to prevent alloreactivity and immunogenicity as well as potentiate robust tumor-specific activity. The problem has been further exacerbated by a gap in the development of renewable sources of precision-engineered T-cells, largely resulting from bottlenecks in manufacturing and facile multiplexed genetic ablation strategies.

#### CRISPR-Cas9 can be used to engineer off-the-shelf allogeneic CAR T-cells

The primary challenge to overcome in the setting of allogenic products is the induction of graft-versus-host-disease (GvHD), where the endogenous donor T-cell receptor recognizes ‘non-self’ surface human leukocyte antigen (HLA) molecules in the patient, eliciting an immune reaction. Healthy donor allogeneic CAR T-cells can be derived from patients’ previous HLA-matched hematopoietic stem cell transplant (HSCT) donor or from gene-edited cells which have been modified to allow them to be given to non-HLA matched subjects. There is increasing enthusiasm for the use of so-called ‘third party CAR T-cells’ (i.e., non-autologous T-cells). An off-the-shelf product would allow one to start with T-cells from healthy donors to create large quantities of universal tumor-specific T-cells that could be used in any patient without the need for HLA matching. This strategy would reduce costs, speed drug administration, and make the T-cell products accessible to lymphopenic and critically ill cancer patients that often do not have sufficient numbers of healthy T-cells for treatment. As proof-of-concept for this approach, CAR T-cells derived from healthy unrelated donors have conferred anti-leukemic efficacy in children and adults with relapsed ALL [102].

Ideally, universal T-cell products should have 3 essential characteristics: 1) resistance to recognition/rejection by host natural killer (NK) cells and T-cells, 2) optimized for potency, persistence and safety, and 3) available as on-demand therapeutics. Without the ability to incorporate such features, large-scale and reproducible low-cost production of high quality, safely engineered CAR T-cells cannot be achieved and the promise of cell therapies will remain elusive to vast populations of cancer patients around the world.

The T-cell receptor α constant (TRAC) locus has been extensively studied as an ideal target for both gene knockout and CAR knock-in [90, 91]. Placing the CAR transgene under control of the endogenous TRAC promotor will drive CAR expression in a stable and robust manner parallel to physiological TCR expression. This will also simultaneously knock out the endogenous TCR, which will eliminate GvHD concerns and allow for an allogenic T-cell product to be generated. Combining the CAR T-cell manufacturing process with simultaneous TCR knockout allows for a streamlined approach to universal CAR T-cell generation **(**Fig. 4b, c**)**.

#### Application of multiplex editing and CRISPR-Cas9 variants can optimize universal CAR T-cell therapy

Successful multiplex CRISPR-Cas9 editing of CAR T-cells has been done with targeting inhibitory genes (e.g., *PDCD1*, PD-1) and those encoding death receptors such as CD95/Fas to prevent TME-mediated inhibition or apoptosis [88]. *TRAC*, *PDCD1 *and *B2M *triple knockout CAR T-cells have demonstrated robust anti-tumor function in in vitro and in vivo models incorporating CD19 and prostate stem cell antigen (PSCA)-directed CARs [86, 87]. In terms of the safety and clinical feasibility of such an approach, we conducted the first-in-human trial of multiplex CRISPR-Cas9 edited (i.e., *TRAC, TRBC* and *PDCD1* knockout) T-cells with a transgenic TCR specific for tumor-associated antigen NY-ESO-1 in patients with myeloma and sarcoma [3]. Transfer of gene-edited TCR-engineered T-cells into patients resulted in durable engraftment with edits at all three genomic loci [3]. Although not involving multiplex gene editing, generation of PD-1 deficient T-cells via CRISPR-Cas9 editing of *PDCD1* was also demonstrated to be feasible in the setting of clinical product scalability, and this product proved to be safe in a phase I trial for advanced non-small-cell lung cancer patients [4]. The results of the above investigations clearly demonstrate the feasibility and short-term safety of treating patients with CRISPR-edited human T-cells. Favorable outcomes from additional studies conducted with larger numbers of patients should hopefully justify more advanced Phase II and Phase III trials in the future (Fig. 4c).

The aforementioned Cas9 variants have shown promise in the pre-clinical setting. For example, primary human T-cells have been modified using base editors for disruption of *TRAC, B2M* and *CIITA* to reduce expression of the endogenous TCR, together with MHC class I and II machinery as well as *PDCD1* to prevent additional inhibition of CAR T-cell effector function [103, 104]. Base editors have also shown promising therapeutic potential for enhancing CAR T-cells for treatment of T-cell acute lymphoblastic leukemia (T-ALL). The CAR antigens for T-ALL are typical pan T-cell markers such as CD7 and CD3, and anti-CD7 CAR T-cells for T-ALL have already been proven safe and efficacious in Phase I clinical trials [105]. Introduction of premature STOP codons into the *CD7* and *CD3* loci of anti-CD7 and anti-CD3 CAR T-cells prevents the induction of CAR-mediated fratricide during the manufacturing and infusion process [106]. dCas9 tethered to transcriptional repressor KRAB targeting the PDCD1 gene is effective in generating anti-HER2 CAR T-cells with low PD-1 expression to confer checkpoint inhibition resistance [107]. Prime editors, while still in their infancy, can efficiently edit the genomes of human cell lines and human primary T-cells [108]. The search-and-replace nature of prime editing also provides a potentially more adaptable method to simultaneously introducing additional complex gene edits into T-cells (e.g., certain gain of function mutations) that can enhance CAR T-cell therapy, but are challenging to achieve with endonuclease-based gene or base editing approaches (Fig. 4c).

The challenges of producing a universal CAR T-cell product from finite numbers of mature, fully differentiated healthy donor T-cells are compounded by the anticipated engineering demands, particularly for solid tumors in which multiple CRISPR-Cas9-based gene edits are needed to be combined with CAR-based recognition to achieve safety and efficacy. For example, in the setting of allogeneic T-cell therapies involving checkpoint blockade, expression of a transgene encoding a synthetic antigen receptor is required, in addition to knockout of several inhibitory molecules (e.g., PD-1, CTLA-4, TIM-3, LAG-3) as well as T-cell receptor (TCR) alpha and beta constant regions (*TRAC* and *TRBC*). As these therapies require several genetic edits within mature T-cells, inefficiencies and combinatorial stochasticity in production result in a final product that typically contains many different populations of cells with variable editing combination efficiencies that need to be evaluated and optimized to increase efficacy [103].

To specifically address cell source material to generate allogeneic products, CAR T-cell therapeutics can be derived from inducible pluripotent stem cells (iPSCs) amenable to genetic modification and differentiation into mature tumor-targeted T-cells. iPSCs are obtained from adult somatic cells by inducing expression of a combination of transcription factors (c-MYC, OCT3/4, SOX2 and KLF4) that transform cells into a pluripotent state [109, 110] (Fig. 4c). Pluripotent stem cells can give rise to the three embryonic germ layers, rendering them capable of differentiating into any specified cell type, with lymphoid lineages stemming from mesoderm progenitors [111, 112]. Because iPSCs are stem-like cells, they can be cultured unlimitedly and indefinitely in vitro under proper conditions which allow for a continuous production of T-cells [111]. Proof-of-concept studies have demonstrated successful generation of CAR T-cells from T lymphocyte-derived iPSCs [113]. However, the use of ‘feeder-free’ culture systems will likely be necessary for clinical-grade scalability and production [114]. Ideal conditions to achieve differentiation of mature T-lymphocytes involve incorporation of thymic niche promoting Notch/TCR signaling, as well as soluble factors such as IL-7, FMS-like tyrosine kinase 3 ligand (FLT3), stem cell factor (SCF), and the chemokines CXCL12 and CCL25 [111, 114].

Because downstream differentiated T-cells are derived from the same iPSC clone, CRISPR-Cas9 editing of iPSCs permits stable and consistent knockdown of factors such as the endogenous TCR and major histocompatibility complex (MHC) genes as well as genes encoding inhibitory molecules such as PD-1 to create unique pools of allogenic T-cell products with superior antitumor functionality, as has been previously seen in edited primary T-cells directed against glioblastoma and acute lymphoblastic leukemia in vivo models [90, 115]. Ideally, this will lead to a bank of selected iPSC lines with a consistent and optimally potent downstream differentiated final product for off-the-shelf therapies (Fig. 4c). The combination of precision iPSC programming using CRISPR-Cas9 and CAR technology sets the stage for the emergence of a new class of renewable and highly controllable off-the-shelf T-cell therapeutics.

## Conclusions

The clinical efficacy of CAR T-cell technology has been proven in the setting of human cancer. However, there are major limitations to accessing this technology. Currently, it is a bespoke product made for individual patients, and therefore, the time to manufacture can prevent access as can the cost. Further, the T-cells used as starting material from patients are likely to have developed cancer associated T-cell dysfunction, which may not be reversible. Advances in CRISPR-Cas9-based genome editing will help address the several current unmet needs in CAR T-cell therapy. Such advances may involve DSB-free genome editing methods like base and prime editing to allow precise and controllable genetic modification in a robust manner. In addition, CRISPR-Cas9-induced multiplex knockout of inhibitory molecules potentiates enhanced CAR T-cell expansion and persistence that may allow for circumvention of T-cell-intrinsic as well as -extrinsic resistance mechanisms operative in both hematopoietic and non-hematopoietic malignancies. Targeted knock-in approaches also have the potential to fine-tune transgene insertion during CAR T-cell engineering to generate effective and potent cell products with temporally regulated effector functions. Finally, procurement of normal donor leukocytes or iPSCs to produce a ‘universal’ CAR T-cell product that can be CRISPR-Cas9 engineered to overcome certain histocompatibility barriers and with enhanced persistence/antitumor function will significantly improve the manufacturing of cellular immunotherapies and therapeutic durability. Development of next-generation therapies with CAR T-cells, involving innovative genetic editing with CRISPR-Cas9 technology, will ultimately improve affordability and reduce off-target toxicities while enhancing antitumor effectiveness.

## Data Availability

Not applicable.

## Abbreviations

ALL: Acute lymphoblastic leukemia
BCMA: B-cell maturation antigen
CAR: Chimeric antigen receptor
CLL: Chronic lymphocytic leukemia
Cas9: CRISPR-associated protein 9
CRISPR: Clustered regularly interspaced short palindromic repeats
CRISPRa: CRISPR activation
CRISPRi: CRISPR interference
CRS: Cytokine release syndrome
dCas9: Catalytically dead Cas9
DLBCL: Diffuse large B-cell lymphoma
DNMTs: DNA methyltransferases
DSB: Double-stranded break
FDA: United States Food and Drug Administration
HDR: Homology-directed repair
HLA: Human leukocyte antigen
HSCT: Hematopoietic stem cell transplant
Indels: Insertions and deletions
iPSCs: Induced pluripotent stem cells
KRAB: Krüppel associated box
MHC: Major histocompatibility complex
nCas9: Nickase Cas9
NK cell: Natural killer cell
NHEJ: Non-homologous end joining
PAM: Protospacer adjacent motif
pegRNA: Prime editing guide RNA
RT: Reverse transcriptase
sgRNA: Single guide RNA
TCR: T-cell receptor
TET: Ten-eleven translocation
TME: Tumor microenvironment
TRAC: T-cell receptor α-chain constant
TRBC: T-cell receptor β-chain constant
